# Identification and prognostic analysis of propionate metabolism-related genes in head and neck squamous cell carcinoma

**DOI:** 10.3389/fonc.2025.1518587

**Published:** 2025-06-12

**Authors:** Shitong Zhou, Yu Jiang, Panhui Xiong, Zhongwan Li, Lifeng Jia, Wei Yuan, Xiufu Liao, Xiang An, Jie Hu, Rui Luo, Hailan Mo, Hongyan Fang, Yucheng Yang

**Affiliations:** ^1^ Department of Otorhinolaryngology, The First Affiliated Hospital of Chongqing Medical University, Chongqing, China; ^2^ Department of Otorhinolaryngology Head and Neck Surgery, Chongqing General Hospital, Chongqing University, Chongqing, China

**Keywords:** propionate metabolism-related genes, head and neck squamous cell carcinoma, prognostic risk model, metabolic reprogramming, immune evasion

## Abstract

**Introduction:**

Head and neck squamous cell carcinoma (HNSCC) is a highly heterogeneous malignancy with poor overall prognosis. Recent studies have suggested that propionate metabolism-related genes (PMRGs) may play key roles in tumor progression and immune regulation, yet their functions in HNSCC remain unclear.

**Methods:**

Transcriptomic data from 502 HNSCC tumor samples and 44 normal tissue samples were obtained from the UCSC Xena database as the training set. Two independent datasets (GSE41613 and GSE6631) from the GEO database were used for validation. Differentially expressed genes (DEGs), key module genes identified via weighted gene co-expression network analysis (WGCNA), and PMRGs were intersected to identify candidate genes. A prognostic model was constructed using Cox regression and LASSO analysis. Immune infiltration, somatic mutations, and drug sensitivity were compared between high- and low-risk groups. Gene expression was further validated by RT-qPCR using clinical samples.

**Results:**

A total of 42 intersecting genes were identified, and four feature genes (PRKAA2, SLC7A5, GRIP2, CHGB) were selected to build the prognostic model. The model effectively stratified patients into high- and low-risk groups with significant survival differences in both the training and validation cohorts. The high-risk group exhibited marked differences in immune cell infiltration, immune checkpoint expression, and cancer immune cycle activity. Mutation burden and drug sensitivity also varied significantly between risk groups. A nomogram combining risk score and pathological N stage showed strong predictive performance.

**Discussion:**

This study highlights the potential role of PMRGs in immune regulation and tumor progression in HNSCC. The proposed four-gene signature provides a novel tool for prognosis prediction and offers new insights for risk stratification and individualized therapy. Further multicenter validation and mechanistic studies are warranted.

## 1 Introduction

Head and neck squamous cell carcinoma (HNSCC) is the sixth most common cancer globally, with 5-year survival rates consistently ranging from 40% to 60% over the past few decades (1–3). Clinically, HNSCC is divided into HPV-positive and HPV-negative subtypes based on the presence of human papillomavirus (HPV), each with distinct etiologies, molecular profiles, therapeutic responses, and prognoses (4). HPV-positive HNSCC typically arises in the oropharynx, is more prevalent among nonsmokers, demonstrates relatively stable molecular features, and responds well to chemoradiotherapy, resulting in a favorable prognosis. In contrast, HPV-negative HNSCC is strongly associated with tobacco and alcohol use, displays considerable molecular heterogeneity, and is linked to poorer outcomes, including increased resistance to treatment and higher rates of local recurrence (4, 5). Current precision medicine strategies for HNSCC face two major challenges: the lack of reliable molecular biomarkers for prognostic prediction and significant individual variability in response to chemotherapeutic agents such as docetaxel and methotrexate (6). These issues highlight the urgent need for further exploration of molecular mechanisms to improve risk stratification and therapeutic approaches.

Recent research has emphasized the pivotal roles of tumor metabolic reprogramming and immune evasion. Metabolic reprogramming, for example, has been shown to influence the expression of immune checkpoint molecules such as PD-L1 (7). Tumor cells can increase PD-L1 expression through the activation of transcription factors like HIF-1α, thus suppressing T cell activity and enabling immune escape (8). Additionally, alterations in short-chain fatty acid (SCFA) metabolism, particularly propionate, have been implicated in tumorigenesis and progression (9, 10). Propionate, a key SCFA produced primarily through gut microbial fermentation of dietary fiber, not only contributes to energy metabolism but also plays pivotal roles in immunomodulation, epigenetic regulation, and cellular signaling (11). Growing evidence suggests that disturbances in propionate metabolism are closely associated with malignant progression and metastasis in various cancers (12). For instance, propionate promotes the differentiation of regulatory T cells (Tregs) and inhibits proinflammatory Th17 cells by activating G-protein-coupled receptors (GPR43/41) and suppressing HDAC activity, thereby fostering an immunosuppressive tumor microenvironment (TME) (13). Moreover, metabolites such as methylmalonic acid (MMA) can induce CD8^+^ T-cell exhaustion and enhance PD-L1 expression, further contributing to tumor immune evasion (14). In colorectal cancer and melanoma, disrupted propionate metabolism has been linked to the polarization of M2-type tumor-associated macrophages (TAMs) and the recruitment of myeloid-derived suppressor cells (MDSCs), suggesting a role in immune escape (15). Despite these findings, the biological functions and clinical significance of propionate metabolism-related genes (PMRGs) in HNSCC remain largely unexplored.

This study identified key genes associated with propionate metabolism in HNSCC and developed a prognostic model based on these genes. A comprehensive analysis of clinical features, immune cell infiltration, immune checkpoint expression, immune cycle dynamics, and drug sensitivity differences between high- and low-risk patient groups was performed. In summary, the findings of this study uncover potential therapeutic targets linked to propionate metabolism in HNSCC and offer novel insights that may aid in the development of precision treatment strategies for this challenging malignancy.

## 2 Materials and methods

### 2.1 Data source and tissues

Transcriptome sequencing data from 502 HNSCC tumor tissue samples and 44 normal tissue samples were retrieved from the UCSC Xena database (https://xenabrowser.net/datapages/) to serve as the training set. Two additional HNSCC datasets (GSE41613 and GSE6631) were sourced from the GEO database (https://www.ncbi.nlm.nih.gov/gds). The validation set included 97 oral tissue samples from patients with HPV-negative HNSCC from GSE41613 (platform GPL570). For expression verification, 22 tissue samples from patients with HNSCC and 22 normal tissue samples from GSE6631 (platform GPL8300) were utilized. A total of 603 PMRGs were obtained from the GeneCards database (https://www.genecards.org/). Real-time quantitative reverse transcription polymerase chain reaction (RT-qPCR) validation was conducted on tumors and adjacent normal tissues from 24 patients at the Department of Otolaryngology, Chongqing General Hospital. Histological evaluation was performed on each sample, and all participants provided written informed consent. The study was approved by the Ethics Committee of Chongqing General Hospital (Approval No. KY S2023-102-01).

### 2.2 Acquisition of intersecting genes

Gene expression data were standardized by converting probe IDs into gene identifiers and eliminating duplicate entries for the same gene in each sample to ensure a single representation per gene. Subsequently, differential expression analysis was performed using the “limma” package (v 3.58.1) (16) in the training set, identifying differentially expressed genes (DEGs) with a threshold of | Fold Change (FC)| ≥ 1 and adj. *p*< 0.05. Weighted gene coexpression network analysis (WGCNA) was conducted using the “WGCNA” package (v 1.70-3) (17) to identify the most relevant modules for HNSCC in the training set. Hierarchical clustering was initially performed to detect outliers, with any identified outlier samples excluded. The optimal soft threshold was determined based on the scale-free fit index (signed *R* ^2^) and average connectivity (targeting a value close to 0). Genes were then grouped into modules using the hybrid dynamic tree-cutting algorithm. The correlation between these modules and the HNSCC phenotype was calculated, and the modules with the strongest correlations were defined as key modules. Genes within these key modules were identified as key module genes. Intersecting genes were derived by overlapping DEGs, key module genes, and PMRGs. To explore the biological functions and pathways involved in the intersecting genes, Gene Ontology (GO) and Kyoto Encyclopedia of Genes and Genomes (KEGG) enrichment analyses were performed using the clusterProfiler package (v 4.2.2) (18). Protein–protein interactions (PPI) among the intersecting genes were assessed using the STRING database (https://cn.string-db.org/).

### 2.3 Prognostic risk model

Univariate Cox regression analysis was performed on the intersecting genes in the training set to calculate the *p*-values, hazard ratios (HRs), and their 95% confidence intervals (CIs) for each gene (*p*< 0.05, HRs ≠ 1). Genes identified by univariate Cox regression were further analyzed using the Least Absolute Shrinkage and Selection Operator (LASSO) with the “glmnet” package (v 4.1-2) (19). Tenfold crossvalidation was conducted using the cv.glmnet function, and candidate genes were selected based on the lambda.min value that minimized the prediction error. These candidate genes were then subjected to multivariate Cox regression analysis (*p*< 0.05) and proportional hazards (PH) testing (*p* > 0.05) to identify feature genes. The risk score for each patient in the training set was calculated using the following formula: $\sum_{n}^{n}=1\left(coefi*Xi\right)$ . The median risk score was used to categorize the samples into high- and low-risk groups. Survival analysis was then conducted, and the Kaplan–Meier (K-M) curve was generated using the “survival” package (v 3.3-1) (20) (*p*< 0.05). Receiver operating characteristic (ROC) analysis was performed using the plotROC package (v 2.3.1) (21), and ROC curves for 1-, 3-, and 5-year survival were plotted, with the area under the curve (AUC) calculated (AUC > 0.7). Additionally, principal component analysis (PCA) was performed to evaluate the discriminative ability of the risk score in the training set. The same methodology was applied to validate the risk model in the validation set. The Wilcoxon test was used to assess differences in the expression of feature genes between HNSCC and control samples in both the training set and the validation set (GSE6631) (*p*< 0.05), with heatmaps generated to visualize the expression patterns.

### 2.4 Relationship between risk scores and clinical characteristics

Differential expression of feature genes across various clinical characteristics and risk groups was analyzed. The distribution of samples among each clinical characteristic group in the two risk groups was also examined. Additionally, differences in risk scores across clinical feature subgroups were evaluated, and survival differences between different risk subgroups within each clinical characteristic subgroup were computed.

### 2.5 Construction and evaluation of the nomogram model

Univariate and multivariate Cox regression analyses, based on risk scores, age, gender, stage, pathological T, pathological N, and grade, were performed using the “survival” package (v 3.3-1) to identify independent prognostic factors. The rms package (v 6.8-1) (21) was then employed to construct a nomogram based on the independent prognostic factors. The nomogram’s predictive performance was assessed using calibration and decision curves.

### 2.6 Differential expression analysis

To explore the differential gene expression between the high- and low-risk groups, differential expression analysis was performed using the DESeq2 package (v 1.34.0) (16) in the training set with the threshold set at |log_2_FC| ≥ 1 and adj. *p*< 0.05. GO and KEGG enrichment analyses were conducted on the DEGs between the two risk groups using the clusterProfiler package (v 4.2.2) (18). Single-sample Gene Set Enrichment Analysis (ssGSEA) for KEGG pathways was performed across all samples in the training set, identifying pathways that differed between the high- and low-risk groups.

### 2.7 Somatic cell mutation, drug sensitivity, immune microenvironment, and immune cycle analyses

Somatic mutations in patients with HNSCC were analyzed and visualized using the maftool package (v 2.10.5). Mutation categories, types, and the frequency of the top 25 mutated genes were examined in both the high- and low-risk groups. Chemotherapeutic agents for HNSCC were obtained from the GDSC database (https://www.cancerrxgene.org). The IC_50_ values for common chemotherapeutic and molecularly targeted drugs in each HNSCC sample were calculated using the R package pRRophetic (v 0.5) (22). Differences in IC_50_ values between the high- and low-risk groups were assessed using the Wilcoxon rank-sum test. Subsequently, ssGSEA of 16 immune cell types, eight immune functions, 19 immune checkpoints, and seven immune cycles was performed for both groups in the training set using the GSVA package (v 1.42.0) (16). The estimate package (v 1.0.13) (18) was used to calculate stromal, immune, and ESTIMATE scores for each HNSCC sample in the training set, and differences in these scores were compared between the high- and low-risk groups.

### 2.8 RNA isolation, RT-PCR, semi-quantitative PCR, and qPCR

Total RNA was extracted from cell lines and tissues using TRIzol Reagent (Invitrogen, Carlsbad, CA, USA) following the manufacturer’s protocol. The RNA was quantified through spectrophotometry and stored at − 80°C. Primer sequences are listed in **Table 1**.

**Table 1:** Primer sequences in the RT-qPCR experiment.

Genes	Forward primer (5*′*–3*′*)	Reverse primer (5*′*–3*′*)
PRKAA2	TCAATCGTTCTGTCGCCA	CGTTAGCATCATAGGAAGGG
CHGB	GACCACCATTCAACCCAC	CCCAACTCTCCTCACTCTG
SLC7A5	GCCGAGGAGAAGGAAGA	TGCCCGAGCCGATAATG
GRIP2	CCCTCGTGTGCTTCATCG	GCTTCCTCCATAGTCCC

For qPCR, SYBR Green (Thermo Fisher Scientific, Hong Kong, China) was used according to the manufacturer’s instructions, with amplification performed on a 7500 Real-Time PCR System (Applied Biosystems, Foster City, CA, USA). GAPDH served as the internal control. Gene expression levels were calculated using the 2^−ΔΔCt^ method, with all samples analyzed in triplicate.

### 2.9 Statistical analysis

Statistical analyses were performed using GraphPad Prism 9.0 (GraphPad Software Inc., CA, USA) and SPSS 23.0 (IL, USA). All experiments were conducted in triplicate, and data are presented as the mean ± standard deviation. Normality and equality of variance were assessed using the Shapiro–Wilk and Levene tests, respectively. For normally distributed data, comparisons between groups were made using Student’s *t*-test, with Welch’s correction for unequal variances. For non-normally distributed data, the Mann–Whitney *U* test was employed. The Wilcoxon rank-sum test was used to compare ssGSEA scores between groups. The Cox regression model was tested for PH assumptions, and survival analysis was conducted using the log-rank test.

## 3 Results

### 3.1 Intersecting genes were related to fatty acid metabolic processes

**Figure 1:**
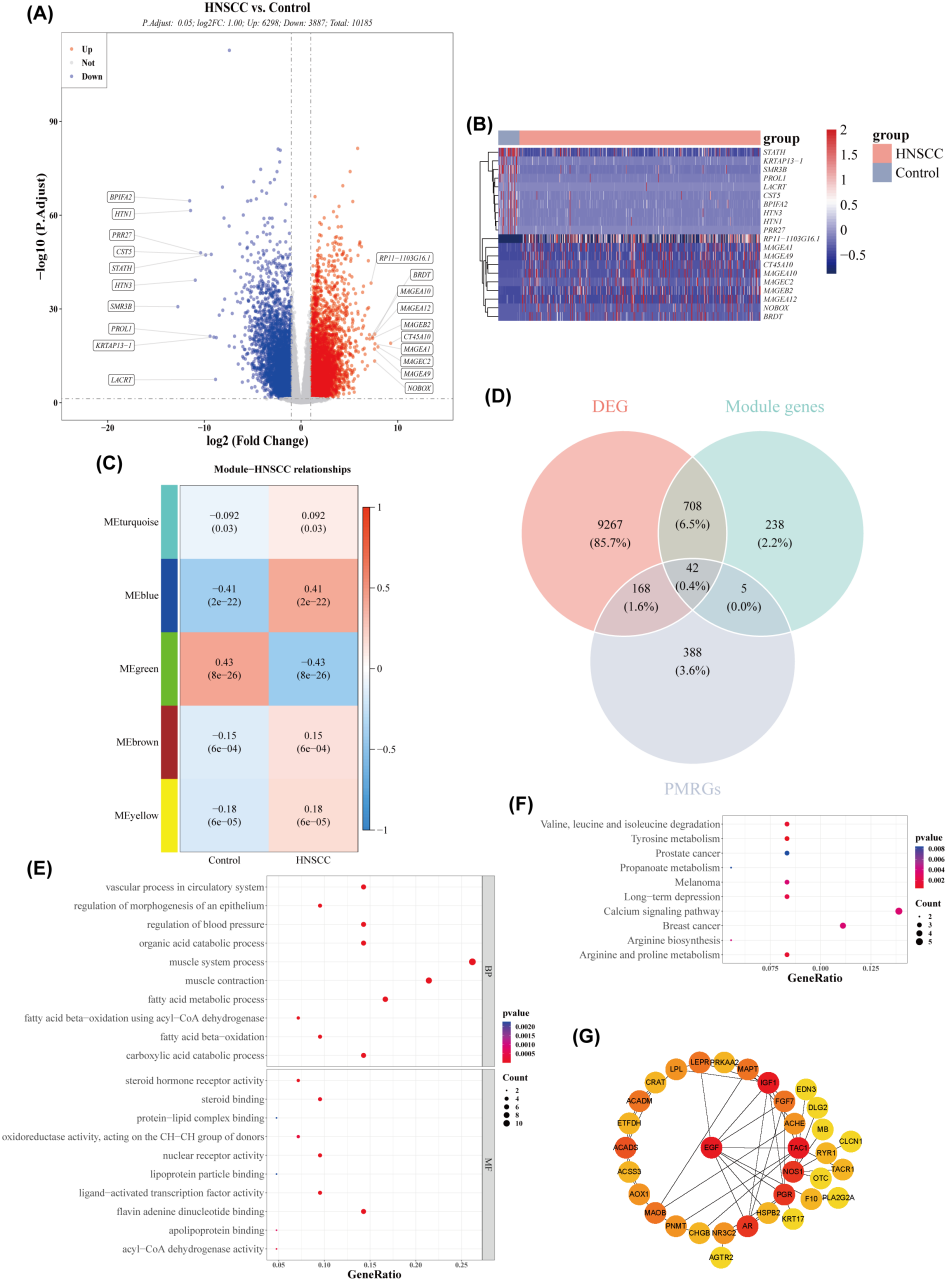
Acquisition of differentially expressed genes (DEGs) and key module genes. **(A)** Volcano plot of DEGs between HNSCC and normal samples (|log_2_FC| > 0.5 and *p*-value< 0.05). **(B)** Heatmap of the top 20 DEGs between HNSCC and normal samples. **(C)** Correlation heatmap between gene modules and disease status. **(D)** Venn diagram showing the overlap of module genes, DEGs, and propionate metabolism-related genes (PMRGs) for screening PMRG-DEGs. **(E)** GO enrichment bubble plot. **(F)** KEGG enrichment bubble plot. **(G)** PPI network.

A total of 10,185 DEGs were identified in the training set, with 6,298 genes upregulated and 3,887 genes downregulated in HNSCC (**Figures 1A, B**). WGCNA revealed the green module, comprising 993 genes, as the most highly correlated with HNSCC (Cor = 0.43, adj. *p* = 2 × 10^−22^) (**Figure 1C**). Forty-two intersecting genes were derived by overlapping the 10,185 DEGs, 993 key module genes, and 603 PMRGs (**Figure 1D**). GO analysis of these intersecting genes highlighted pathways such as fatty acid metabolic processes and protein-lipid complex binding (**Figure 1E**). KEGG pathway analysis further identified involvement in pathways such as alanine, leucine, and isoleucine degradation (**Figure 1F**), suggesting that these genes may influence HNSCC by modulating fatty acid metabolism. To investigate potential gene interactions, a PPI network was constructed. Genes such as ACADM and ACADS, ACHE and MAPT, as well as ACSS3 and AOX1, showed significant interactions (**Figure 1G**).

### 3.2 Prognostic risk models were constructed based on PRKAA2, SLC7A5, GRIP2, and CHGB

**Figure 2:**
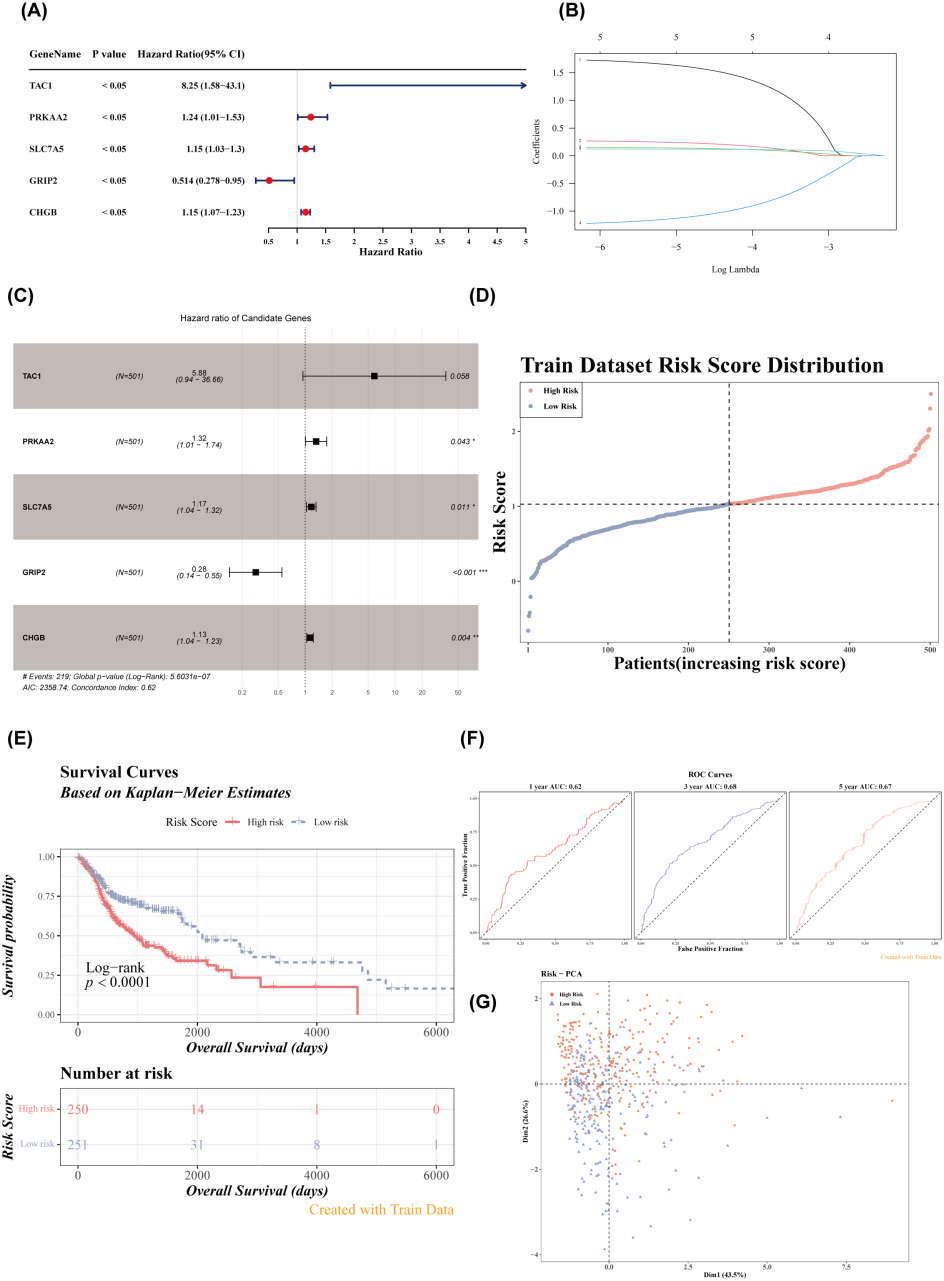
Risk model construction and evaluation in the training set. **(A)** Forest plot of univariate Cox analysis. **(B)** Regression coefficient-lambda plot. **(C)** Forest plot of multivariate Cox analysis. **(D)** Risk score distribution in the training dataset. **(E)** Survival curves of high- and low-risk groups in the training set. **(F)** ROC curves for 1-, 3-, and 5-year survival based on the training set. **(G)** PCA dendrogram.

**Figure 3:**
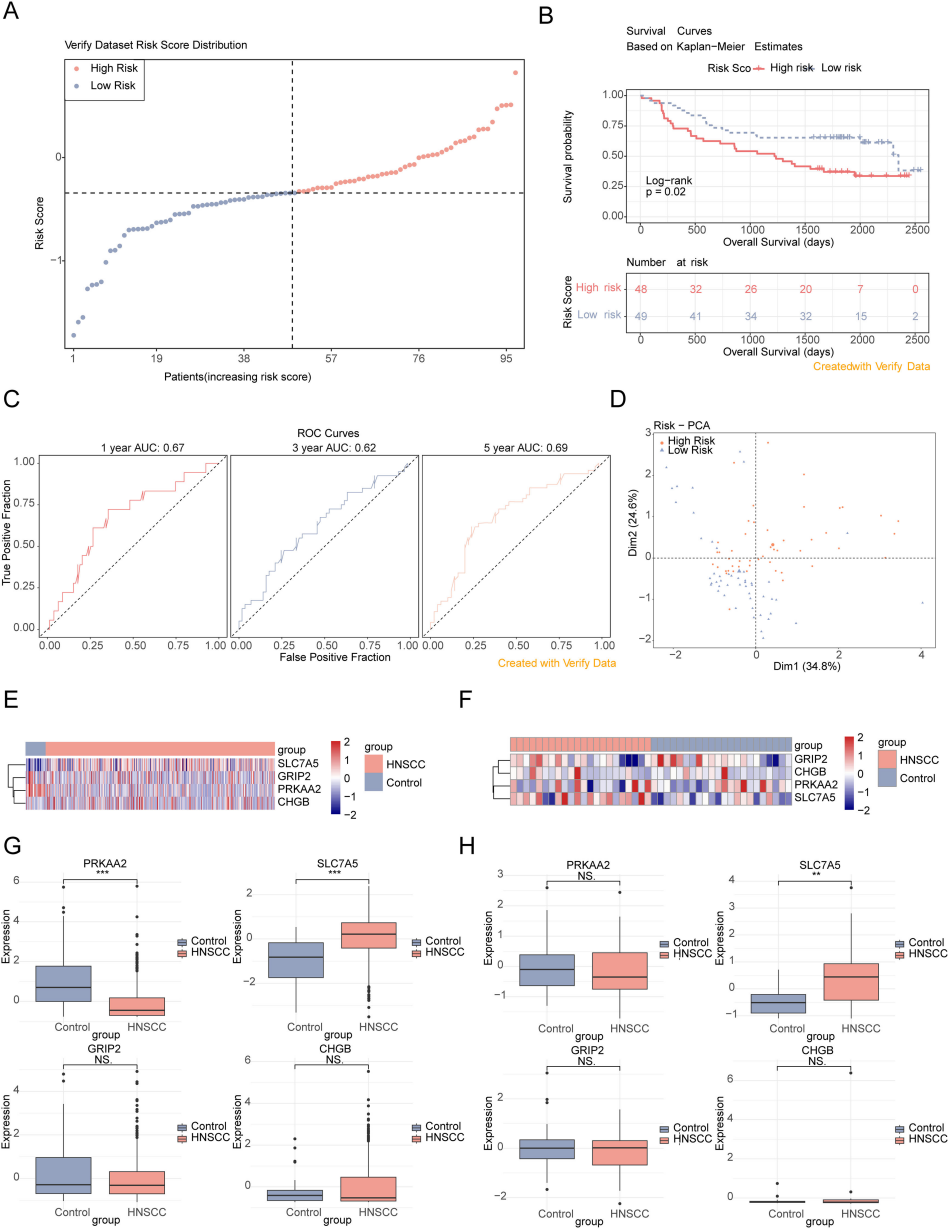
Validation of the risk model in the verification set. **(A)** Risk score distribution in the verification dataset. **(B)** Survival curves of high- and low-risk groups in the verification set. **(C)** ROC curves for 1-, 3-, and 5-year survival based on the verification set. **(D)** PCA dendrogram. **(E, F)** Heatmaps of model gene expression (training and verification datasets). **(G, H)** Box plots of feature gene expression in the training set and verification set (GSE6631). ns, *p* > 0.05; ^**^ *p*< 0.01; ^***^ *p*< 0.001.

Univariate Cox regression analysis identified five genes (TAC1, PRKAA2, SLC7A5, GRIP2, CHGB) with *p*< 0.05 and HR ≠ 1 (**Figure 2A**). Four feature genes (PRKAA2, SLC7A5, GRIP2, CHGB) were selected through LASSO and multivariate Cox regression analysis (**Figures 2B, C**). Based on these feature genes, risk scores for patients with HNSCC in the training set were calculated. Patients were stratified into high- (*n* = 250) and low-risk (*n* = 251) groups based on the median risk score. As the risk score increased, mortality rates also increased (**Figure 2D**), with patients in the low-risk group exhibiting significantly longer survival (Log-rank test *p*< 0.0001) (**Figure 2E**). The AUC values for the 1-, 3-, and 5-year ROC curves of the risk model were all greater than 0.6, indicating strong model performance (**Figure 2F**). PCA demonstrated that the risk scores effectively distinguished between samples in the training set (**Figure 2G**). External validation in the GSE41613 dataset yielded consistent results with the training set (**Figures 3A–D**). The Wilcoxon test confirmed that the expression trends of feature genes in control and disease samples were consistent across both datasets, with SLC7A5 showing significant upregulation in HNSCC samples (*p*< 0.01) (**Figures 3E–H**).

### 3.3 Nomogram diagram could effectively predict the risk profile of patients with HNSCC

**Figure 4:**
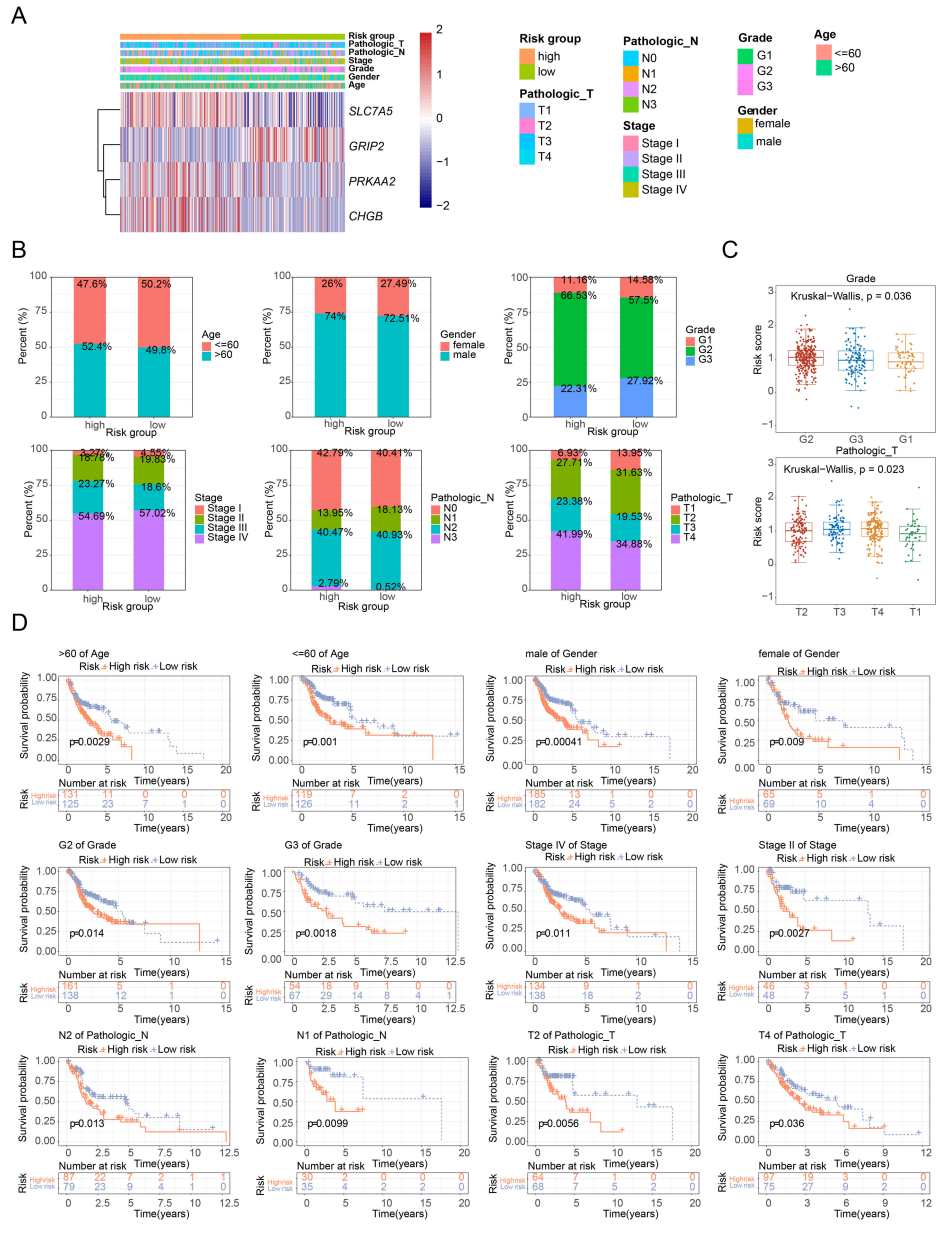
Analysis of risk scores across clinical subgroups. **(A)** Heatmap of model gene expression across different clinical groups. **(B)** Distribution of clinical characteristics in high- and low-risk groups. **(C)** Boxplot of risk scores among different clinical characteristic subgroups. **(D)** Survival curves of high- and low-risk groups across different clinical characteristic subgroups.

The expression of feature genes across different subgroups is shown in **Figure 4A**. The distribution of clinical characteristics in the high- and low-risk groups is presented in **Figure 4B**. Risk scores significantly differed between tumor grading and pathological stage T subgroups, but not between age, gender, tumor grading, tumor stage, and pathological stage N subgroups, indicating that risk scores are more closely associated with tumor grading and pathological stage T (**Figure 4C**). Significant survival differences between high- and low-risk groups were observed across 12 subgroups: age (≤ 60, > 60), gender (women, men), tumor grade (G2, G3), tumor stage (stage II, stage IV), pathological stage N (N1, N2), and pathological stage T (T2, T4) (**Figure 4D**).

**Figure 5:**
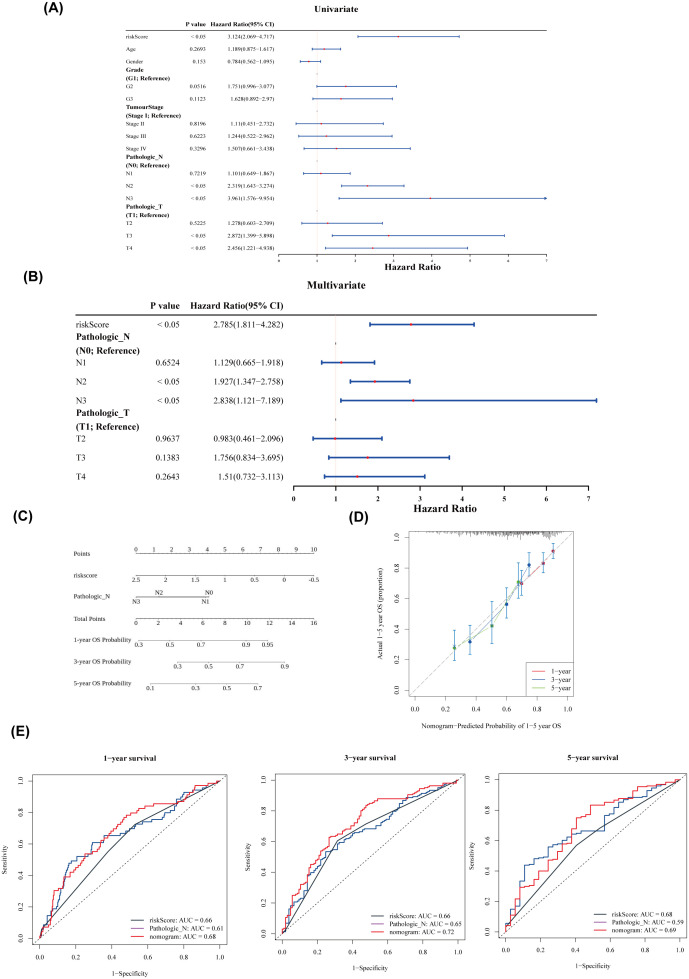
Nomogram construction and evaluation. **(A)** Forest plot of univariate Cox analysis. **(B)** Forest plot of multivariate Cox analysis. **(C)** Nomogram of independent prognostic factors. **(D)** Predicted probabilities of 1–5-year overall survival (OS) based on the nomogram. **(E)** ROC curves for 1-, 3-, and 5-year survival.

Univariate and multivariate Cox regression analysis identified two independent prognostic factors: pathological stage N and risk score (**Figures 5A, B**). A nomogram was constructed based on these two factors (**Figure 5C**). The calibration curves for 1-, 3-, and 5-year survival showed slopes close to 1 (**Figure 5D**), indicating that the nomogram has high predictive accuracy. Furthermore, the 1-, 3-, and 5-year ROC curves for the nomogram demonstrated AUC values greater than 0.6 (**Figure 5E**), suggesting excellent prediction performance. In conclusion, the nomogram developed in this study exhibits favorable accuracy in predicting 1-, 3-, and 5-year overall survival (OS) in patients with HNSCC.

### 3.4 DEGs were related to immunity

**Figure 6:**
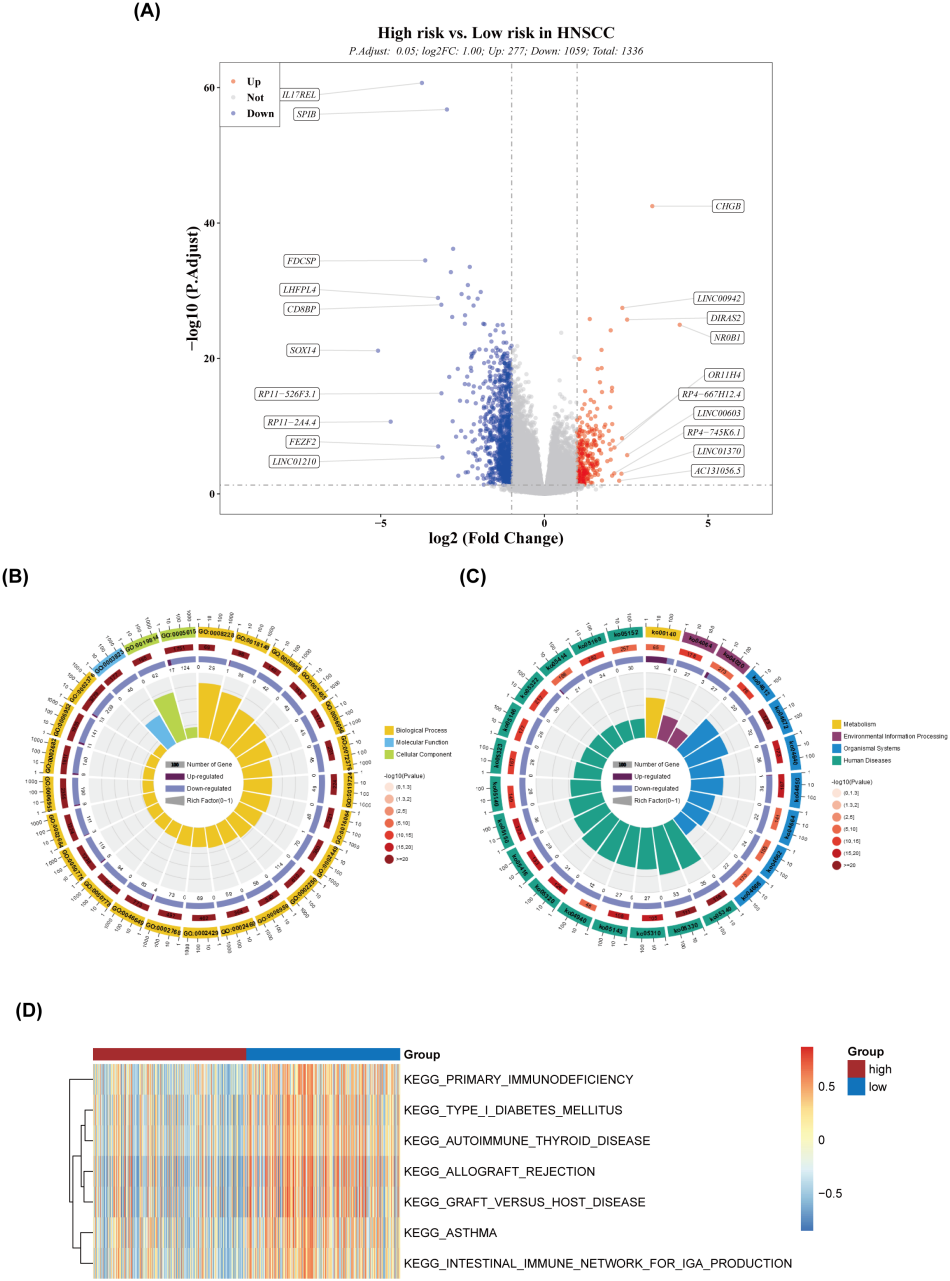
Differential expression and pathway analysis. **(A)** Volcano plot of differentially expressed genes. **(B)** Circular plot of GO enrichment. **(C)** Circular plot of KEGG enrichment. **(D)** Heatmap of ssGSEA scores for the pathways.

A total of 1,336 DEGs were identified between the high- and low-risk groups, with 277 genes upregulated and 1,059 genes downregulated in the high-risk group (**Figure 6A**). GO analysis of these DEGs highlighted pathways such as adaptive immune response and immune system processes (**Figure 6B**). KEGG pathway analysis identified involvement in pathways such as primary immunodeficiency and the intestinal immune network for IgA production (**Figure 6C**), suggesting that these DEGs may influence risk scores through modulation of immune responses. The ssGSEA scores for seven of the 186 pathways showed significant differences between the two groups (**Figure 6D**), with the high-risk group exhibiting generally lower scores in these pathways.

### 3.5 High-risk high-mutation rate

**Figure 7:**
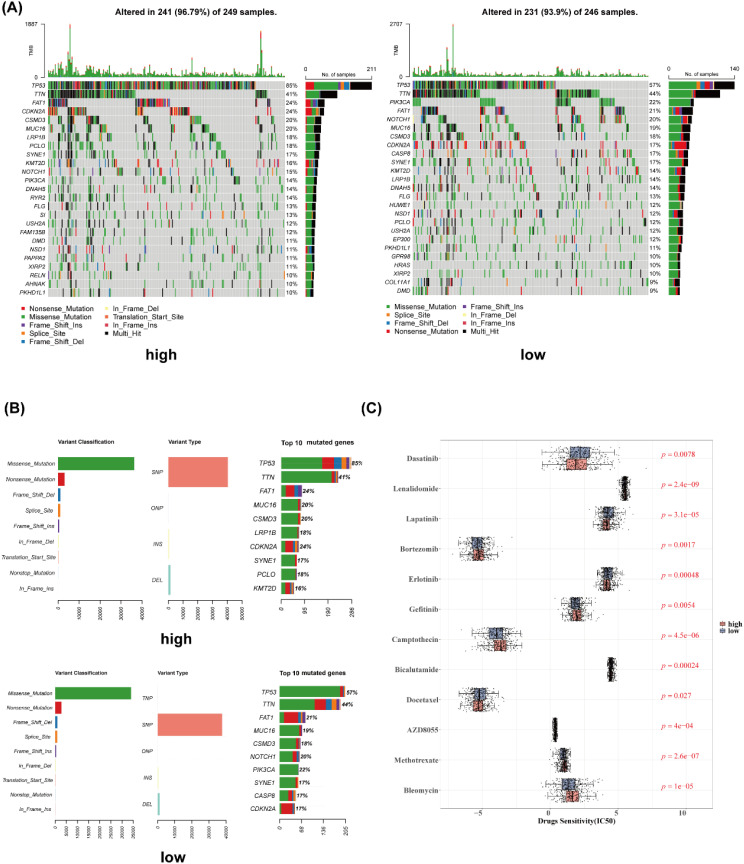
Gene mutation analysis and drug sensitivity. **(A)** Waterfall plot of gene mutation analysis (high- and low-risk groups). **(B)** Gene mutation cartogram (high- and low-risk groups). **(C)** Boxplot of differential drug sensitivity analysis.

In this study, 96.79% of samples in the high-risk group and 93.9% of samples in the low-risk group exhibited mutations in the top 25 most frequently mutated genes (**Figure 7A**). The most common mutation type was missense mutation (SNP), with TP53 showing the highest mutation frequency across samples (**Figure 7B**). Additionally, 12 drugs displayed significant differences in sensitivity between the high- and low-risk groups, such as dasatinib, lenalidomide, and lapatinib (**Figure 7C**).

### 3.6 Significant differences in immune cells, immune checkpoints, and immune cycles

**Figure 8:**
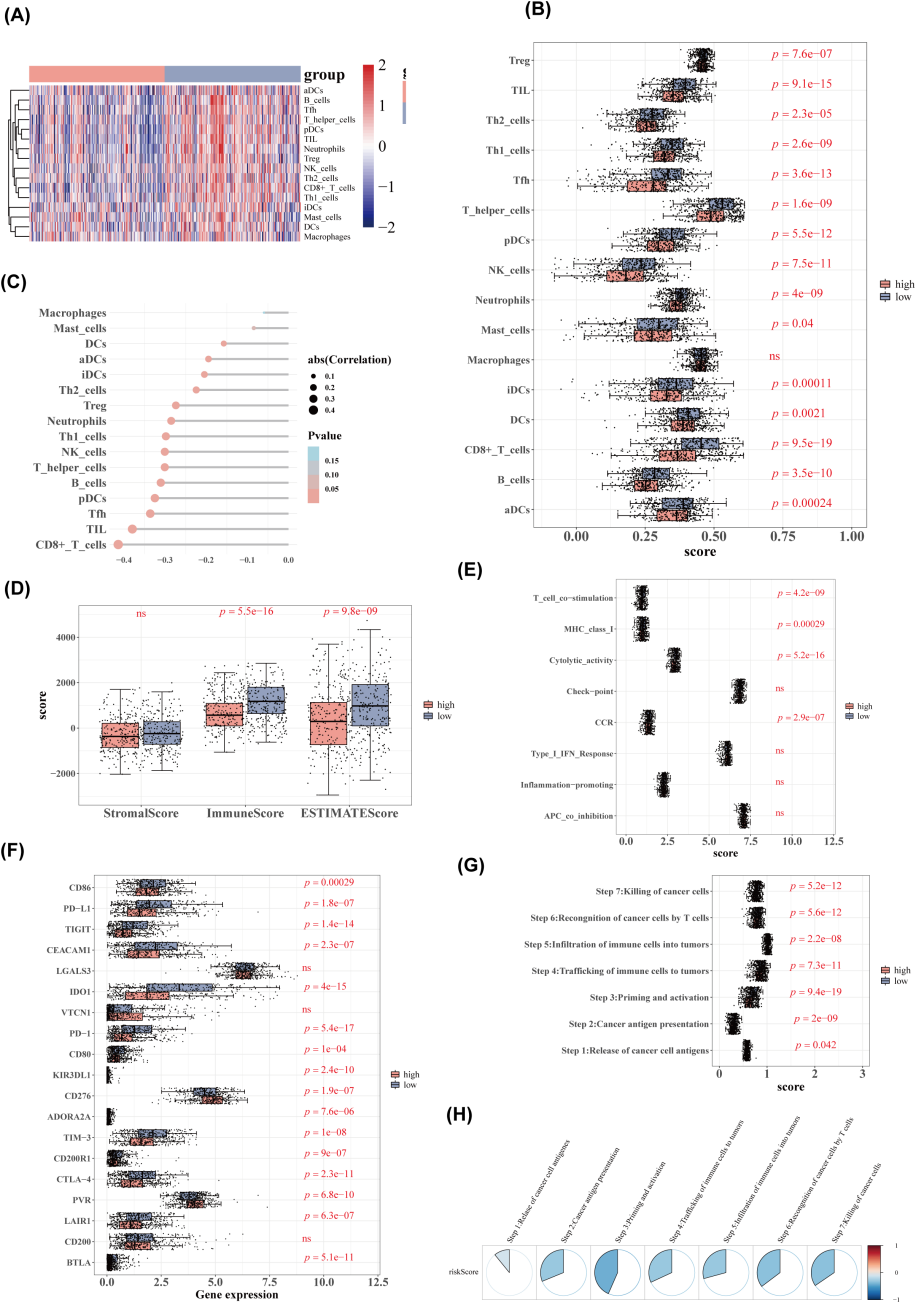
Immune cell and immune function analysis. **(A)** Heatmap of immune cell ssGSEA scores. **(B)** Boxplot of immune cell ssGSEA scores. **(C)** Lollipop diagram of correlation analysis between risk scores and immune cell scores. **(D)** Boxplot of stromal, immune, and ESTIMATE scores between high- and low-risk groups. **(E)** ssGSEA scores for immune function between high- and low-risk groups. **(F)** Boxplot of immune checkpoint inhibitor expression. **(G)** Boxplot of cancer immune cycle scores. **(H)** Correlation analysis between cancer immune cycle scores and risk scores.

The heatmap of ssGSEA scores for 16 immune cell types is shown in **Figure 8A**. Except for macrophages, the remaining 15 immune cell types exhibited significant differences in scores between the high- and low-risk groups (**Figure 8B**). There was a negative correlation between risk scores and the scores of immune cells, with the strongest correlation observed between risk scores and CD8^+^ T cells (**Figure 8C**). Significant differences in immune scores and ESTIMATE scores were found between the two groups, while stromal scores showed no significant differences (**Figure 8D**). The ssGSEA scores for four immune functions were significantly different between the groups (**Figure 8E**), and 16 immune checkpoints also exhibited significant differences (**Figure 8F**). All seven cancer immune cycle scores differed significantly between the high- and low-risk groups (**Figure 8G**). Furthermore, these cancer immune cycle scores were negatively correlated with risk scores, with STEP 3 showing the strongest correlation (**Figure 8H**).

### 3.7 Validation of the mRNA expression of four genes (PRKAA2, GRIP2, CHGB, SLC7A5) in HNSCC

**Figure 9:**
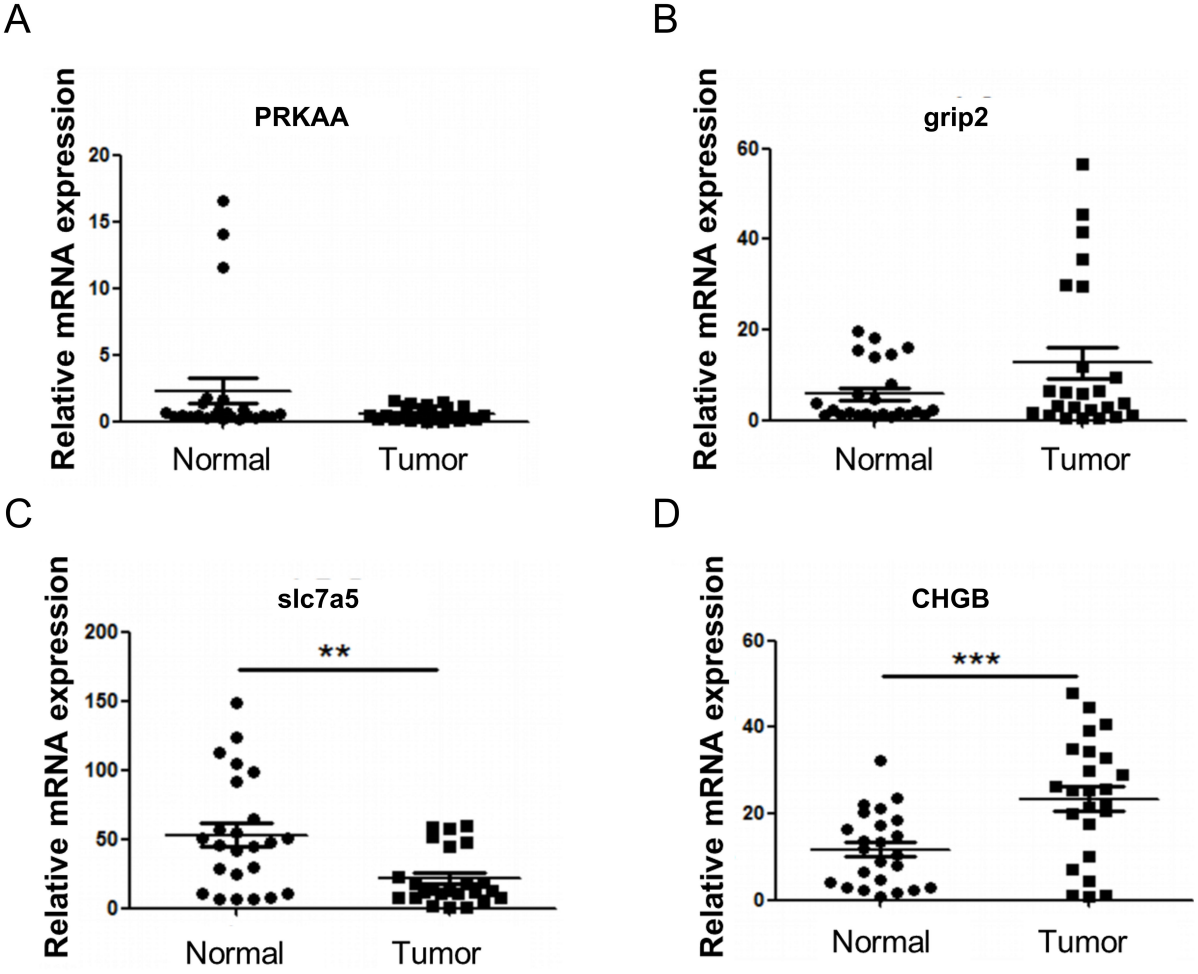
RT-qPCR verification of model genes. **(A)** Expression of PRKAA. **(B)** Expression of GRIP2. **(C)** Expression of CHGB. **(D)** Expression of SLC7A5. Compared with Normal, **P<0.01, ***P<0.001.

To validate the expression changes of the feature genes in HNSCC, 24 pairs of tumor and adjacent noncancerous tissues were collected, and qPCR was performed for verification. The results showed no significant expression changes for PRKAA and GRIP2 in head and neck tumors (**Figures 9A, B**), whereas CHGB exhibited a noticeable upregulation, and SLC7A5 showed downregulation in head and neck tumors (**Figures 9C, D**).

## 4 Discussion

HNSCC is a highly heterogeneous malignancy whose development is strongly linked to HPV infection and immunometabolic reprogramming within the TME (23). Recent studies have highlighted the involvement of SCFAs, particularly propionate, as microbial metabolites that regulate energy metabolism and influence tumor progression through epigenetic modifications and immunomodulatory pathways (24). However, the exact mechanisms of PMRGs in HNSCC remain poorly understood. In this study, four characteristic genes associated with propionate metabolism in HNSCC—PRKAA2, SLC7A5, GRIP2, and CHGB—were identified through bioinformatics analysis, and their potential roles were explored, providing new theoretical insights for future research on HNSCC.

PRKAA2, also known as AMPKα2, encodes the catalytic α2 subunit of AMP-activated protein kinase (AMPK) (25). It regulates glucose metabolism, which affects tumor cell growth and energy supply (26). Notably, PRKAA2 expression is significantly elevated in hepatoblastoma (HB), where it acts as an oncogenic factor by promoting cell proliferation and inhibiting ferroptosis (27). In non-small cell lung cancer (NSCLC), PRKAA2 enhances tumor growth and suppresses ferroptosis via the SLC7A11/GSH/GPX4 pathway (28). These findings suggest that PRKAA2 may similarly influence tumor cell proliferation and survival in HNSCC.

SLC7A5 (LAT1) facilitates the cellular uptake of neutral amino acids, including leucine and glutamine (29). Its transport of leucine activates the mTORC1 signaling pathway, thereby promoting protein synthesis to support rapid tumor cell proliferation (30). Tumor cells can modulate SLC7A5 expression to alter immune cell function and evade immune surveillance (31, 32). Li et al. identified SLC7A5 as a potential prognostic biomarker in HNSCC associated with immune infiltration (33), suggesting that therapeutic targeting of SLC7A5 may offer a novel strategy for treatment.

GRIP2 encodes a PDZ domain-containing protein that binds GluR2 to anchor AMPA receptors within neuronal signaling complexes, playing pivotal roles in synaptic transmission and plasticity (34). Given the frequent dysregulation of signaling pathways in cancer cells (35), GRIP2 may influence HNSCC progression by modulating key tumorigenic pathways. Interestingly, GRIP2 has been linked to variations in innate CD8^+^ T cells (36), suggesting its potential immunomodulatory effects in HNSCC progression. Thus, GRIP2 may regulate both tumor signaling pathways and immune cell function, making it a promising therapeutic target.

CHGB is a highly conserved eukaryotic protein involved in secretory regulation (37). While CHGB genetic variants have been associated with cardiovascular disease risk (38) and the protein regulates ion channels to maintain secretory granule homeostasis (37), its role in cancer remains poorly understood and warrants further investigation.

Drug sensitivity analysis identified 12 compounds, including dasatinib, lenalidomide, and lapatinib, with significantly different IC_50_ values between high- and low-risk groups, suggesting their potential clinical applications. Dasatinib, a multi-target tyrosine kinase inhibitor, may enhance treatment response in high-risk patients by inhibiting SRC family kinases and exerting immunomodulatory effects (39–41). The immunomodulator lenalidomide could improve the TME and increase sensitivity to chemoradiotherapy (42, 43). Lapatinib, an oral tyrosine kinase inhibitor targeting the EGFR/HER2 pathways, may provide precision therapy for specific molecular subtypes (44). These observed differences in drug sensitivities support the rationale for molecular classification and personalized treatment strategies in HNSCC. Validation through *in vitro* experiments and clinical cohorts is essential, alongside exploration of combination therapies with existing treatments, such as immune checkpoint inhibitors, to refine and optimize precision treatment regimens.

Significant differences in the expression of 16 immune checkpoint genes, including PD-L1, were identified between risk groups. Previous studies have shown that HNSCC cells often overexpress PD-L1, which binds to PD-1 on T cells, thereby suppressing their activation and function, enabling immune evasion (45–47). This immunosuppression is a key mechanism driving HNSCC progression (48). Furthermore, PD-L1 overexpression is associated with poorer prognosis in patients with HNSCC (49), likely due to reduced survival rates from PD-L1-mediated immune suppression. The elevated expression of PD-L1 in high-risk patients observed in this study supports these immune escape mechanisms and offers valuable insights for understanding prognostic differences and developing novel immunotherapies.

In summary, this study identified four characteristic genes associated with propionate metabolism through bioinformatics analysis and established a risk model based on these genes. These findings provide new insights for prognostic assessment and the development of innovative therapeutic strategies for HNSCC. However, several limitations must be acknowledged. The current sample size necessitates further validation through multicenter studies with larger cohorts to confirm the clinical applicability of the model. Additionally, while these metabolic genes have been identified as potential therapeutic targets, their precise mechanisms in modulating the immune microenvironment require further functional studies and clinical trials. Future research should refine this risk stratification system and investigate metabolism-targeted combination therapies to develop more precise treatment strategies for patients with HNSCC.

## Data Availability

The raw data supporting the conclusions of this article will be made available by the authors, without undue reservation.
